# Comparison of the Effects of Inorganic or Amino Acid-Chelated Zinc on Mouse Myoblast Growth *in vitro* and Growth Performance and Carcass Traits in Growing-Finishing Pigs

**DOI:** 10.3389/fnut.2022.857393

**Published:** 2022-04-07

**Authors:** Lingyu Zhang, Qiuping Guo, Yehui Duan, Xue Lin, Hengjia Ni, Chuanshe Zhou, Fengna Li

**Affiliations:** ^1^Hunan Provincial Key Laboratory of Animal Nutritional Physiology and Metabolic Process, Key Laboratory of Agro-ecological Processes in Subtropical Region, Institute of Subtropical Agriculture, Chinese Academy of Sciences, Beijing, China; ^2^College of Advanced Agricultural Sciences, University of Chinese Academy of Sciences, Beijing, China; ^3^National Engineering Laboratory for Rice and By-product Deep Processing, Central South University of Forestry and Technology, Changsha, China; ^4^Guangzhou Tanke Bio-tech Co., Ltd., Guangzhou, China

**Keywords:** organic zinc, growth performance, C2C12 cell line, growing–finishing pigs, cell cycle

## Abstract

This study aimed to investigate the effects of the supplementation of different sources of zinc on mouse myoblast growth *in vitro* and the growth performance and carcass traits in growing-finishing pigs. In the *in vitro* trial, 25 or 75 mM zinc sulfate (ZnSO_4_), methionine-chelated zinc (ZnMet), and glycine-chelated zinc (ZnGly) were co-cultured with the myoblast during proliferation and differentiation. The results showed that the amino acid-chelated zinc supplementation, especially ZnMet, enhances cell proliferation and differentiation in mouse myoblast, and regulates the distribution in S and G2/M phases (*P* < 0.05). Meanwhile, the protein expression levels of the mammalian target of rapamycin pathways were up-regulated after treatment with 25 μM ZnMet (*P* < 0.05), which is consistent with the results of the enriched Kyoto Encyclopedia of Genes and Genomes (KEGG) pathway in the transcriptome analysis. In the *in vivo* trial, 27 Duroc × (Landrace × Large White) pigs with an initial average weight of 31.62 ± 0.36 kg were divided into three groups with nine replicates per treatment. The dietary treatment groups were as follows: (1) ZnSO4 group, basal diet +75 mg/kg ZnSO4; (2) ZnMet group, basal diet +75 mg/kg ZnMet; and (3) ZnGly group, basal diet +75 mg/kg ZnGly. The whole trial lasted for 75 days. Increased final body weight, average daily gain, and decreased F/G were noted in the ZnMet group (*P* < 0.05). Moreover, the ZnMet group had higher carcass weight and loin eye area (*P* = 0.05). The ZnMet and ZnGly group both had lower serum total protein (*P* < 0.05), while the ZnMet group had higher serum alkaline phosphatase (*P* < 0.05). Also, the addition of ZnMet showed higher concentrations of zinc and iron in muscle, kidney, and serum (*P* < 0.05), improving the deposition and availability of micronutrients. In conclusion, amino acid-chelated zinc, particularly ZnMet, had the best effect, which could improve growth *in vitro* and increase growth performance while boosting bioavailability in growing-finishing pigs, ultimately, enhancing muscle mass, providing a theoretical basis and guidance for the future use of amino acid-chelated zinc to effectively replenish energy in animal nutrition and production.

## Introduction

Zinc is an integral part of more than 200 enzymes in mammals, directly involved in various physiological processes, such as DNA and protein synthesis, proliferation, differentiation, and apoptosis in the entire life (1–4). Indeed, the deficiency of zinc could cause numerous metabolic disorders, such as oxidative stress or immune system-related diseases, and may even increase COVID-19 infectious risk (5–7). In recent years, zinc sources have experienced the development process from inorganic zinc to organic zinc form, including amino acid-chelated zinc (8). Among them, several studies have shown that amino acid-chelated zinc has higher biological efficacy, utilization rate, and better stability, which could improve growth performance and reduce environmental pollution (8, 9).

Amino acid-chelated zinc can facilitate the absorption of trace minerals effectively as it can be absorbed directly as a whole through the cell membrane into the plasma, while inorganic zinc must be chelated with amino acids or other substances, such as coenzyme, to be absorbed (8, 10, 11). Meanwhile, amino acid-chelated zinc is supposed to protect zinc from any reaction with phytates, also leading to a higher bioavailability (12). However, in terms of inorganic zinc, the formation of complexes of ZnSO_4_ with phytates could reduce zinc absorption in the gut (12), suggesting that amino acid-chelated zinc can not only be absorbed quickly but also shows unique nutritional characteristics and biological functions different from the inorganic zinc. However, the underlying mechanisms remain unknown.

When nutrients are available, the mechanistic target of rapamycin (mTOR) is activated to coordinate eukaryotic cell growth and metabolism (13). It is reported that zinc supplementation affected the PI3K/AKT/mTOR pathway by stimulating the phosphorylation of AKT and the downstream target mTOR, thus in turn, improving protein synthesis and deposition (14). Moreover, the transcriptome analysis showed that methionine-chelated zinc (ZnMet) increased anabolism by integrating the mTOR signaling pathway in juvenile yellow catfish (9). Besides, it is indicated that zinc is an essential nutrient for proliferation because it may regulate the cell-cycle (15, 16). Of note, one of the zinc finger proteins, Roma, is an essential regulator of the cell cycle and is required to maintain proliferation (17). Another metabolic response is that zinc supplementation also regulates mitochondrial functions, including oxidative phosphorylation (OXPHOS), thus driving ATP synthesis (18). Accordingly, it seems reasonable to hypothesize that the amino acid-chelated zinc (ZnMet or glycine-chelated zinc, ZnGly) may also activate the mTOR pathway, simultaneously regulating the cell cycle and OXPHOS, even more efficiently than inorganic zinc, which in turn improves metabolic pathways and systemic growth.

The development of animal models has led to research on the effects of the amino acid-chelated zinc on growth responses, but so far there have been only a few studies on growing-finishing pig models. In fact, growing-finishing pigs and humans have many similarities in anatomy and physiology (19). Therefore, our research can guide the efficient use of amino acid-chelated zinc in human nutrition and animal production in the future. The study aimed to compare the effects of inorganic zinc (zinc sulfate, ZnSO_4_) and amino acid-chelated zinc (ZnMet and ZnGly) on the growth performance, absorption, and metabolism in C2C12 mouse myoblast cell line and growing-finishing pigs, and also reveal the underlying mechanisms of the role of amino acid-chelated zinc in energy metabolism and growth responses.

## Materials and Methods

### Cell Culture

Mouse C2C12 cell lines, sourced from American Type Culture Collection (ATCC), were cultured in high glucose (25 mM) Dulbecco's modified Eagle's medium (DMEM) containing 10% (v/v) fetal bovine serum (FBS) (Gibco, Grand Island, NY, USA) in a 5% CO_2_ incubator at 37°C. Once the myoblasts had reached about 90% confluency, the growth media was changed to differentiation media supplemented with 2% (v/v) horse serum (Gibco) for 8 days, as described before (20). ZnSO_4_, ZnMet, and ZnGly (TANKE, Guangzhou, China) were co-cultured with the cells during proliferation and differentiation.

### Cell Growth Characteristics (CCK-8)

C2C12 myoblasts were plated in 96-well plates. The proliferation of the cells was measured using a commercial cell counting kit, CCK-8 (Dojindo, Osaka, Japan), following the manufacturer's specifications. Absorbance was detected at a wavelength of 450 nm (BioTek Instruments, Winooski, USA), and the results were expressed as the optical density (OD_450_).

### Total RNA Extraction and Detection

A total of 12 samples [4 treatments (control, ZnSO_4_, ZnMet, and ZnGly) × 3 biological replications] were used for transcriptome analysis. Total RNA was extracted using a TIANGEN RNAprep Pure kit (TIANGEN, catalog # DP441), according to the manufacturer's instructions. RNA concentrations were measured using a Qubit^®^ RNA Assay Kit in a Qubit^®^ 2.0 Fluorimeter (Life Technologies, CA, USA). The assessment of the RNA integrity number (RIN) was performed using an RNA Nano 6000 Assay Kit and the Agilent Bioanalyzer 2100 system (Agilent Technologies, CA, USA).

### Gene Sequencing

After the total RNA was qualified, quantified, purified, and fragmented into small pieces, first-strand cDNA and second-strand cDNA were synthesized. After the cDNA was incubated with A-tailing mix and RNA Index Adapters for end-repair, the cDNA fragments obtained were amplified by PCR. The products were purified, dissolved, and validated on an Agilent Technologies 2100 Bioanalyzer for quality control. The double-stranded PCR products were then heated, denatured, and circularized with a splint oligo sequence to obtain a final library of single-stranded circular DNA (ssCir DNA). The final library was amplified with phi29 to make a DNA nanoball, which was loaded into a patterned nanoarray. Paired-end reads of 100 base pairs were generated on a BGISEQ-500 platform [Beijing Genomic Institute (BGI), Shenzhen, China]. Finally, the gene expression in each group and the differences among groups were analyzed using a database for mice built by the BGI (Shenzhen). The gene expression levels were calculated with RSEM (v1.2.12).

### Quantitative Real-Time PCR Validation

Quantitative real-time PCR (qPCR) was performed to confirm the transcriptome results. Total RNA (1.5 μg) was reverse transcribed to cDNA with the SMARTerTM PCR cDNA Synthesis Kit (Clontech Laboratories Inc, USA), as per the manufacturer's instructions, and qPCR was carried out using an ABI Prism^®^ 7500 (Bio-Rad, USA) with SYBR Premix Ex TaqII (TAKARA, Japan) by following the instructions of the operation manual. Each reaction was replicated three times per biological replicate, with glyceraldehyde 3-phosphate dehydrogenase (GAPDH) gene as the internal reference gene. The relative quantification of the transcript levels was performed using the 2^−ΔΔCT^ method. The 20 μl reaction volumes contained 2 μl cDNA, 0.4 μl L/R primers (10 μM), 10 μl 2 × qPCR SYBR Premix Ex TaqII, and 7.2 μl double-distilled water. Sequences of the primers for qPCR used in this study are listed in Table 1.

**Table 1:** Real-time PCR primer sequence.

**Gene^a^**	**Size (bp)**	**Forward (5^′^-3^′^)**	**Reverse (5^′^-3^′^)**
ZIP4	260	GAGGAGGAGGTGGGCGTTGGT	AGTTCCGGGGTCTCCTCTGCC
ZIP6	124	GAGGATGTGGAGAGCAAGAAGCAG	GGGAGGGCTCTTGGGAGTCG
ZIP8	88	CTAACGGACACATCCACTTCGA	CCCTTCAGACAGGTACATGAGCTT
ZIP9	100	CCAGAAACAGCAAGGCCCAGCA	TAGAGGCTGCTGCTCCCAAAGC
ZIP11	119	GCTCCAAGGTTACAGCTCCGTGGT	CTAAGATCCGCCTCTGCCCGCT
ZIP14	296	AACGAGCACCATCACGGGCA	TGGAGCCCATCGCTCAGGGT
ZnT1	138	GCTGTGCTTCGCCATCCTGCT	TGATGGTGGAACAGACAGAGCCC
ZnT2	102	GCACCTTCCTCTTCTCCATCCTGGT	TGAAGTCCACGCCTTTGGGAGT
ZnT4	161	AGTGTTGGTGTGCTTATAGCTGCAT	TGGCTTGGTACACCTTCCAGGAT
ZnT5	130	TCACGGCCATTCACACCATGC	GCTGCCCAGTGTGTCTGCCAA
ZnT6	90	CCCTGATGACGTTTGGCACCATGT	ACTGACCGATAACATGAGGTGGTGT
ZnT7	116	CACGGACATTCTCATGGCTCTGGC	GCTCCGTGCTTGGCTTCGTGA
MyoG	485	GCAGGCTCAAGAAAGTGAATG	CACTTAAAAGCCCCCTGCTAC

a *ZIP, ZRT, IRT-like protein; ZnT, zinc transporter; MyoG, myogenin*.

### Cell Cycle Analysis by Flow Cytometry

After treatment, about 1 × 10^6^ cells were rinsed and harvested, then re-suspended in a phosphate-buffered saline (PBS)/ethanol mixture (30/70%), and incubated in PBS containing 200 μg/ml propidium iodide (PI) (Sigma) and 1 mg/ml RNase (Sigma). The samples were then analyzed using a FACScan flow cytometer (Becton Dickinson, San Jose, USA). The fluorescence intensities of the cells stained with PI were monitored at 630 nm. The flow cytometry data were analyzed by FlowJo software.

### Western Blotting Analysis of mTOR Pathway

The cells were rinsed twice using PBS, harvested, pelleted by centrifugation, and lysed in RIPA buffer (150 mM NaCl, 1% Triton X-100, 0.5% sodium deoxycholate, 0.1% SDS, 50 mM Tris-HCl at PH 7.4), combined with a protease inhibitor cocktail and phosphatase inhibitors. Soluble proteins (20–30 μg) were subjected to SDS-PAGE, then transferred to a PVDF membrane, blocked with 5% non-fat milk in TBS-with 0.05% Tween-20 for 1 h, and incubated overnight with primary antibodies, including phosphorylated eukaryotic translation initiation factor 4E binding protein 1 (p-4EBP1) (bs-14550R, Bioss, Beijing, China), 4EBP1 (60246-1-Ig, Proteintech, USA), p-Seventy kilodalton ribosomal protein S6 kinase 1 (P70S6K1) (ab2571, Abcam, UK), P70S6K1 (14485-1-AP, Proteintech, USA), P-mTOR (ab109268, Abcam, UK), and mTOR (ab32028, Abcam, UK), followed by horseradish peroxidase-linked secondary antibodies. The protein bands were visualized using a chemiluminescent reagent (Pierce, Rockford, IL) with a digital luminescent image analyzer LAS-1000 (Fujifilm, Japan). The resultant signals were quantified using Alpha Imager 2200 software (Alpha Innotech Corporation, San Leandro, CA) and normalized against the internal control, GAPDH (10494-1-AP, Proteintech, USA).

### Animal Experiment

#### Experimental Design

A total of 27 Duroc × (Landrace × Large White) crossbred castrated boars with an average weight of 31.62 ± 0.36 kg (mean ± standard error of mean, SEM) were allocated to three diets according to the initial body weight (BW) in a complete randomized block design. Each treatment has 9 replicates (pens). Each pen was 2.1 m long and 1.2 m wide with a 50% slatted floor. Pigs were fed *ad libitum* and had free access to clean drinking water. In these three treatments, the pigs were fed an inorganic zinc control diet (ZnSO_4_) and two amino acid-chelated zinc diets (ZnMet and ZnGly), and the total content of zinc in the three treatments were equal, which was 75 mg/kg (Table 2). The whole experiment lasted for 75 days until the average final BW of pigs was 95 kg. This study complied with Chinese guidelines on experimental protocols and animal welfare and was approved by the Animal Protection Committee of the Institute of Subtropical Agriculture, Chinese Academy of Sciences.

**Table 2:** Ingredients and nutritional composition of basic diets.

**Ingredients (%)**	**30–60 kg**	**60–90 kg**
Corn	58.00	67.00
Soybean meal	29.00	23.76
Wheat bran	7.80	6.00
Soybean oil	1.55	0.88
Lys	0.19	0.01
CaHPO_4_	0.69	0.50
Limestone	0.87	0.55
Salt	0.30	0.30
Premix^a^	1.00	1.00
Total	100.00	100.00
DE (MJ/kg)^b, c^	14.20	14.20
CP	18.27	16.30
SID Lys	0.97	0.72
SID Met + Cys	0.57	0.50
SID Thr	0.61	0.56
SID Trp	0.17	0.17
Total Ca	0.60	0.52
Total P	0.51	0.45

a *Supplied per kg of diet: vitamin A, 15,000 IU; vitamin D_3_, 3000 IU; vitamin E, 40 IU; vitamin K_3_, 4 mg; vitamin B_1_, 3 mg; vitamin B_2_, 10 mg; vitamin B_6_, 4 mg; vitamin B_12_, 0.03 mg; biotin, 0.2 mg; folic acid, 2 mg; niacin, 35 mg; D-calcium pantothenate, 20 mg; Cu (as copper sulfate), 15 mg; Fe (as ferrous sulfate), 80 mg; Mn (as manganese oxide), 15 mg; Zn (as ZnSO_4_, ZnMet, and ZnGly), 75 mg; I (as potassium iodide), 0.5 mg; and Se (as sodium selenite), 0.3 mg*.

b *DE, digestible energy; CP, crude protein; SID, standardized ileal digestibility*.

c *Calculated value for DE and amino acids, and analyzed values for other nutrients*.

#### Chemical Analysis of Diets

The crude protein (CP) content was measured as described before (21). Briefly, 0.2 g of the sample was accurately weighed in a tapered bottle together with 8 ml of hydrochloric acid and left at room temperature overnight. Then the tapered bottles were placed on a muffle until the liquid turned transparent. The digested samples were moved to a 100-ml volumetric flask. After cooling down, they were diluted with double steaming water to the volume of 100 mL and mixed. Then 8 ml of the mixture was transferred to a 10-mL centrifuge tube and placed on a flow injection analyzer (SEAL, Germany) to be detected.

The concentrations of calcium (Ca), phosphorus (P), and zinc were determined as followed (22, 23): in brief, 20 ml of sulfuric acid (18 mol/L) and 2.5 mL of nitric acid (HNO_3_) (14 mol/L) were added to 0.5 g of the sample. Solutions were heated from 100 to 200°C for 30 min in a block digestion system equipped with a system to trap nitrous gases (Behr K 20 L, Behr Labor-Technik GmbH, Düsseldorf, Germany). After cooling to 100°C, 2.5 ml of HNO_3_ was added. After heating from 225 up to 300°C for 75 min and subsequent cooling to room temperature, the solutions were filled with double-distilled water to a volume of 500 mL and filtered. The Ca, P, and zinc concentrations of the solutions were measured using an inductively coupled plasma (LCP) atomic absorption spectrometer (NOVAA350, Jena, Germany).

#### Growth Characteristics and Carcass Traits

**F**eed intake was recorded every day, and the BW was recorded on day 1 and day 75 as the initial BW (initial BW) and final BW (final BW), respectively. The average daily feed intake (ADFI) was the total feed intake divided by the number of days, and the average daily gain (ADG) was the total weight gain divided by 75 days. The ratio of ADFI to ADG (F/G) was used as the feed conversion ratio.

At the end of the experiment, pigs were slaughtered by electrocution (250 V, 0.5 A, for 6–8 s). Carcass traits were analyzed using slaughter segmentation, as described before (24). After slaughter, carcass weight was recorded immediately. The perirenal fat around the kidney was separated and weighed. The dressing percentage was calculated by dividing the carcass weight by **BW**. Backfat thickness and loin eye area were measured at the 6th and the 7th rib.

#### Serum Biochemical Indicators

After slaughter, blood samples were collected from the pigs. Serum was separated and used to determine the concentration of serum biochemical indicators, as mentioned before (24). The concentrations of total protein (TP), albumin (ALB), alkaline phosphatase (ALP), blood urea nitrogen (BUN), glucose (GLU), triglycerides (TG), cholesterol (CHOL), low-density lipoprotein (LDL)-cholesterol, high-density lipoprotein (HDL)-cholesterol, and blood ammonia (NH_3_) were analyzed. These indicators were all tested by commercial kits (Leadman Biotech Limited, Beijing, China) and biochemical analytical instruments (Beckman CX4; Beckman Coulter, Germany).

#### Iron, Zinc, and Copper Content in Serum, Urine, and Tissues

About 3 g of the frozen dry samples of muscle, heart, liver, spleen, lungs, and kidneys were weighed in tapered bottles, soaked overnight with 1% HNO_3_, rinsed with double steamed water, slowly 30 ml of HNO_3_ was added and soaked overnight. At the same time, 1 ml serum or urine, 8 ml 1% HNO_3_, and 1ml double-steamed water were taken in the tapered bottles. Then the tapered bottles were heated until the liquid disappeared. The solids in the cone bottles were dissolved with 1% HNO_3_ and then transferred to a 25-ml capacity tube. The tube was sealed with a sealing film, mixed upside down, and let stand. The mixture was filtered with medium-speed qualitative filter paper, collected in a 10-ml centrifuge tube, and spectrally measured with LCP atomic absorption spectroscopy (NOVAA350, Jena, Germany). To determine the precision of the analysis, all the instrument parameters were optimized daily, as described previously (25).

### Statistical Analysis

All physiological data are presented as the mean of three replicates *in vitro* and nine replicates *in vivo* among the samples with different forms of zinc treatments. One-way analysis of variance (ANOVA) was performed with Duncan's multiple-range test to determine the significant differences between the treatments using IBM SPSS 26.0 statistical software. Significant differences (*P* < 0.05) and a trend of difference (*P* = 0.05) among the treatments are indicated by different letters.

## Results

### Morphological and Physiological Responses of C2C12 Cells to Different Zinc Supplementation

**Figure 1:**
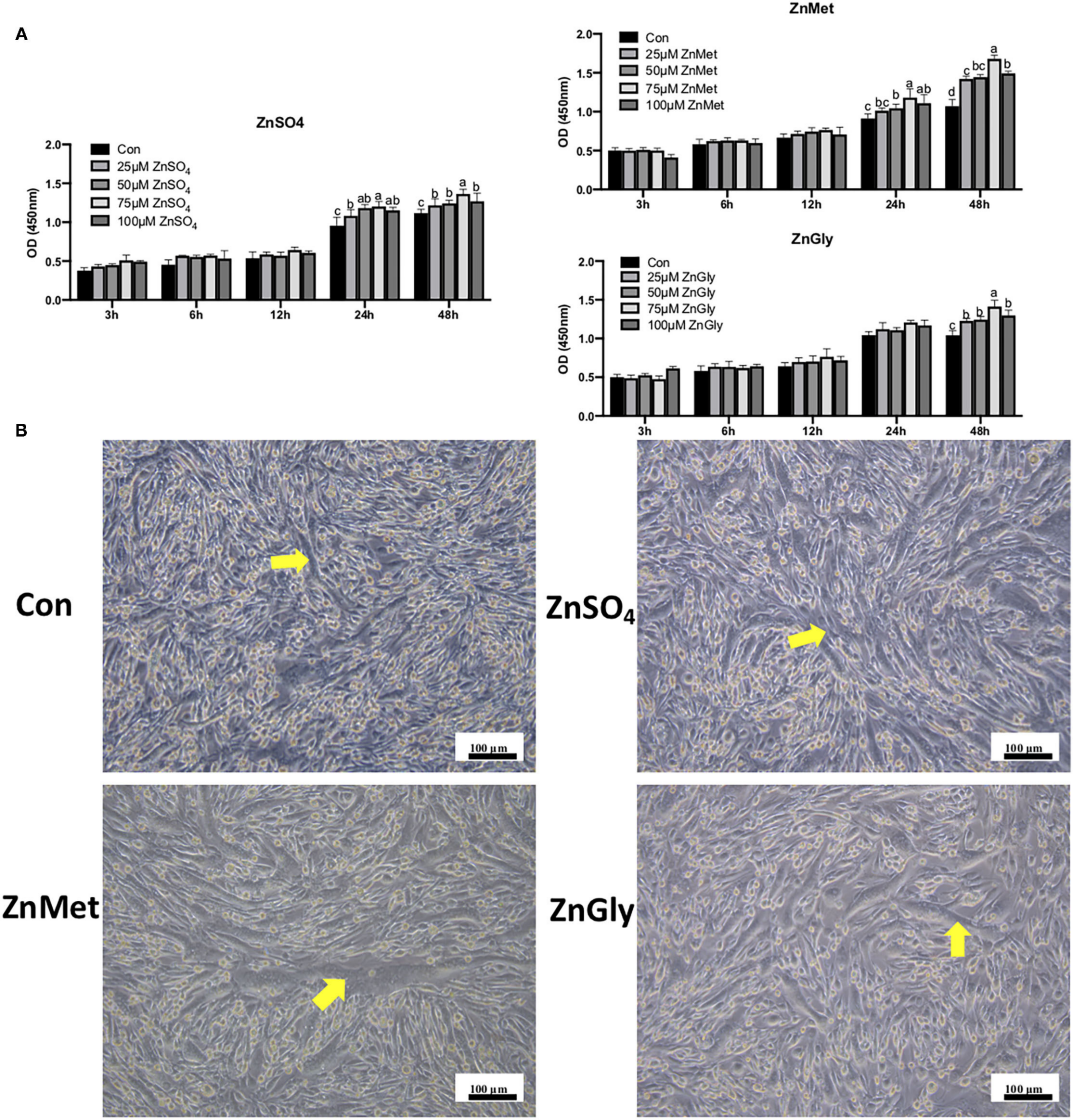
CCK-8 **(A)** in cell proliferation and morphological responses in cell differentiation **(B)** of C2C12 cell line by adding different zinc sources.

As presented in Figure 1A, ZnSO_4_, ZnMet, and ZnGly were diluted to different concentrations (0, 25, 50, 75, and 100 μM) and treated with C2C12 cells for 3, 6, 12, 24, and 48 h, respectively. The cell viability (OD_450_ value) showed that different concentrations of ZnSO_4_, ZnMet, and ZnGly from 3 to 12 h had no effect on cell growth. However, a significant effect was observed after 24 h of treatment in ZnSO_4_ and ZnMet groups, and this effect was observed after 48 h in all three groups. In the three treatments, the OD_450_ value increased when zinc concentration increased from 25 to 75 μM and decreased when OD value increased 75–100 μM. In particular, 25 μM was found to markedly augment C2C12 myoblasts after 48 h compared to the control (*P* < 0.05), but had no significant difference at 50 μM. Therefore, the treatment of myoblast cells with 25 μM ZnSO_4_, ZnMet, and ZnGly for 48 h during proliferation was used for subsequent analysis.

After 8 days of induced differentiation, the myotubes were arranged in a bundle that can be clearly seen in four groups, especially in the ZnMet group. As shown in Figure 1B, the number of myotubes in the control group is significantly less than ZnSO_4_, ZnMet, and ZnGly groups. Meanwhile, the arrangement of the myotubes in the ZnMet and ZnGly groups is more regular than the ZnSO_4_ group. It can be seen that ZnMet and ZnGly can significantly promote differentiation and the formation of myotubes in C2C12 cells.

### Overview of the Data Generated From RNA-seq

After filtering the raw data, a total of 823,297,754 clean reads (100.00% of the raw reads) were obtained from RNA-seq, ranging from 64,624,786 to 71,492,136, among the 12 samples. The mean data size was 6.86 Gb, and the Q20 was higher than 97.85% (Supplementary Table 1), which indicates that RNA-seq data were of high quality. The correlation coefficients of the three replications for each treatment were between 0.995 to 0.998 (Supplementary Figure 1), which were greater than the value of 0.9 recommended to indicate good experimental replication and no contamination in the experiment. The clean reads generated by RNA-Seq were mapped to the reference sequencing, and the mapping rate varied from 80.43 to 84.11% for all samples (Supplementary Table 2).

### Identification of Differently Expressed Genes and Kyoto Encyclopedia of Genes and Genomes Analysis

**Figure 2:**
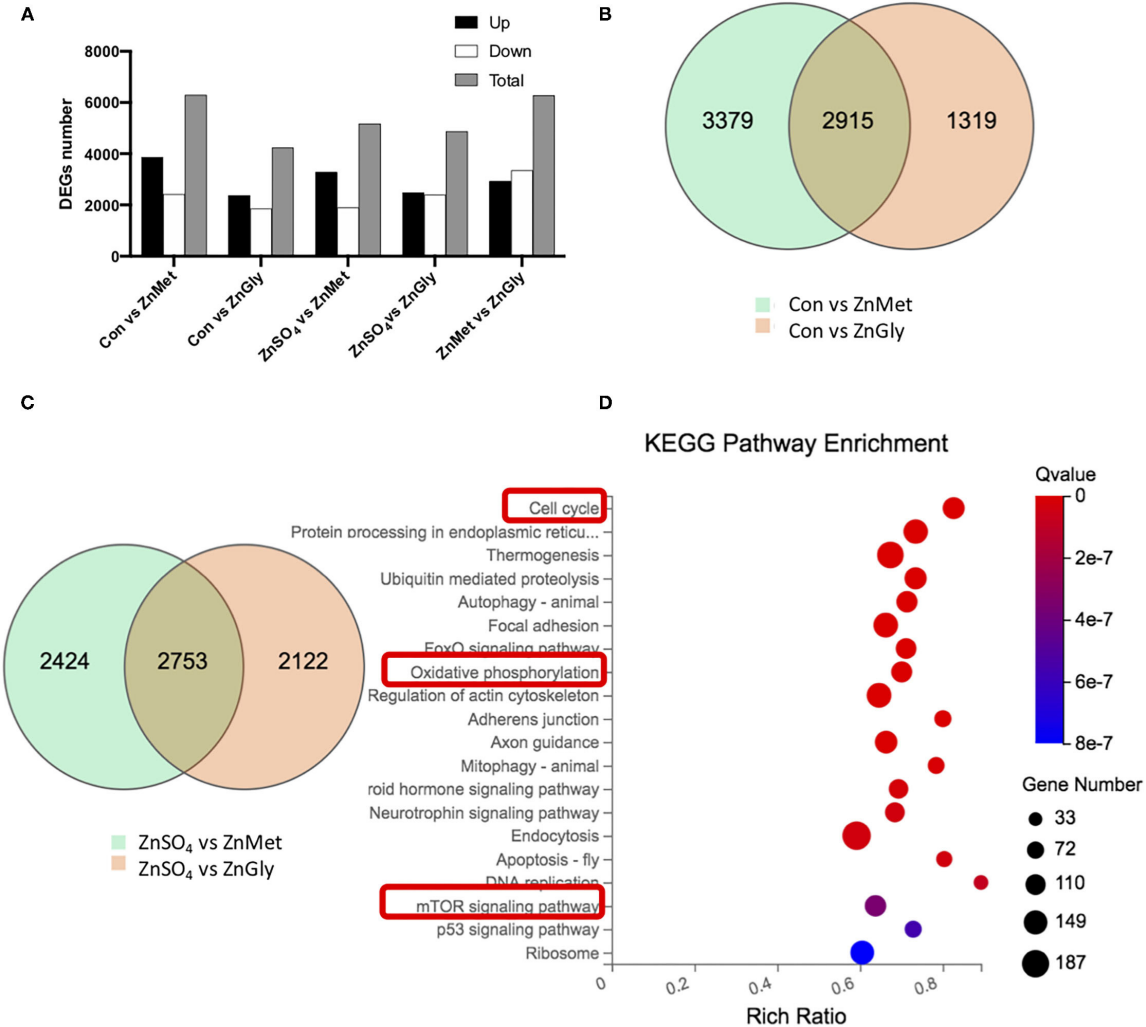
The numbers **(A)**, Venn diagram **(B,C)** of differentially expressed genes (DEGs), and top Kyoto Encyclopedia of Genes and Genomes (KEGG) pathways of DEGs **(D)** in C2C12 cell line by adding different zinc sources.

Analysis of DEGs, when compared to the control group, revealed that there were 6,294 and 4,234 DEGs identified for the ZnMet and ZnGly treatments, respectively. Meanwhile, on using ZnSO_4_ as the control for the ZnMet and/or ZnGly treatments, 5,177 and 4,875 DEGs were identified for the ZnMet and ZnGly treatments, respectively. Compared to the ZnMet treatment, there were 6,279 DEGs identified for the ZnGly treatment. On comparing with either the control group or the ZnSO_4_ group, ZnMet treatment had the highest number of DEGs. There were more up-regulated than down-regulated genes, except in the ZnMet vs. the ZnGly group, where the number of down-regulated genes was 1.14 times that of up-regulated genes (Figure 2A). The Venn diagram showed that when compared to the control group, 2,915 DEGs were commonly induced by both the ZnMet and ZnGly treatments, while 2,753 DEGs were commonly induced by both the ZnMet and ZnGly treatments when compared to the ZnSO_4_ group, which accounted for 25.2 and 39.7% of the total number of DEGs, respectively (Figures 2B,C).

To further investigate the function of DEGs implicated in the metabolic responses of amino acid-chelated zinc, the genes were analyzed using the KEGG database. The enrichment of the KEGG pathway was analyzed to predict potential pathways. A total of 9,171 DEGs were mapped and 8,605 DEGs were assigned to 255 KEGG pathways in this study. The top 20 enriched pathways are shown in Figure 2D. Among these pathways, the cell cycle (ko04110) pathway had the lowest q-value, as well as a high rich ratio and gene number. Meanwhile, the oxidative phosphorylation (ko00190) pathway and the mTOR signaling pathway (ko04150) were also significantly enriched. It is assumed that amino acid-chelated zinc regulated the cell cycle and oxidative phosphorylation, simultaneously activating the mTOR pathway, thus improving the metabolic pathways and systemic growth.

### qPCR Validation

**Figure 3:**
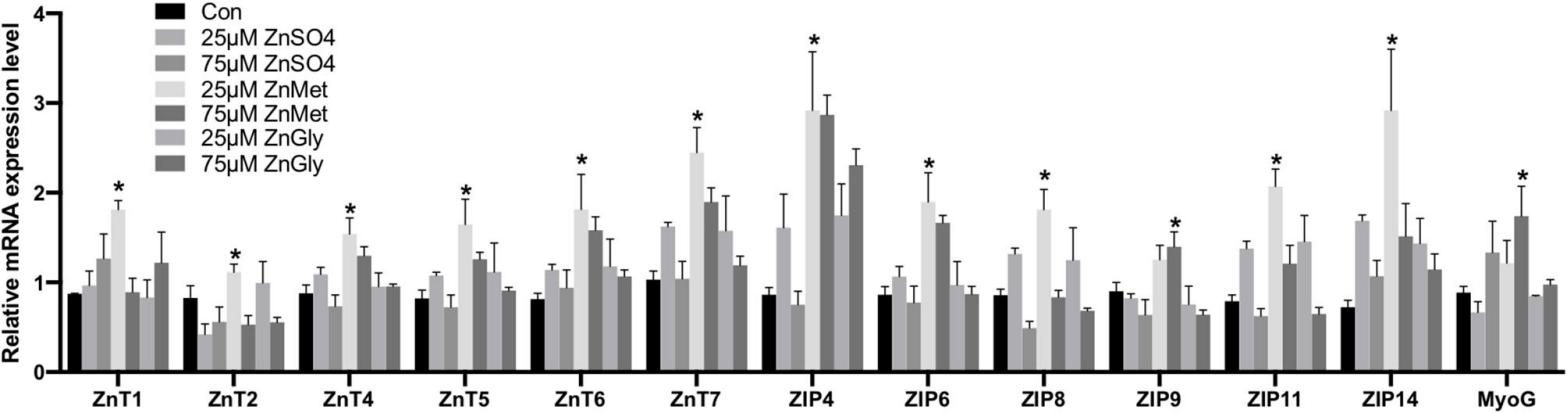
The mRNA expression levels of the key genes of C2C12 cells by adding different zinc sources. The * above the bar indicates the significant difference (*P* < 0.05) among different treatments, *n* = 9.

As shown in Figure 3, 25 μM ZnMet significantly increased relative mRNA levels of ZnT1, ZnT2, ZnT4, ZnT5, ZnT6, ZnT7, ZIP4, ZIP6, ZIP8, ZIP11, and ZIP14 in myoblasts as compared to control or ZnSO_4_ (*P* < 0.05)_._ Meanwhile, 75 μM ZnMet significantly increased relative mRNA levels of ZIP9 and MyoG in myoblasts as compared to control or ZnSO_4_ (*P* < 0.05)_._ The relative expression of 13 genes detected by RT-qPCR had the same tendency of changes with the RNA-seq, indicating the reliability of the RNA-seq data.

### Cell Cycle Analysis

**Figure 4:**
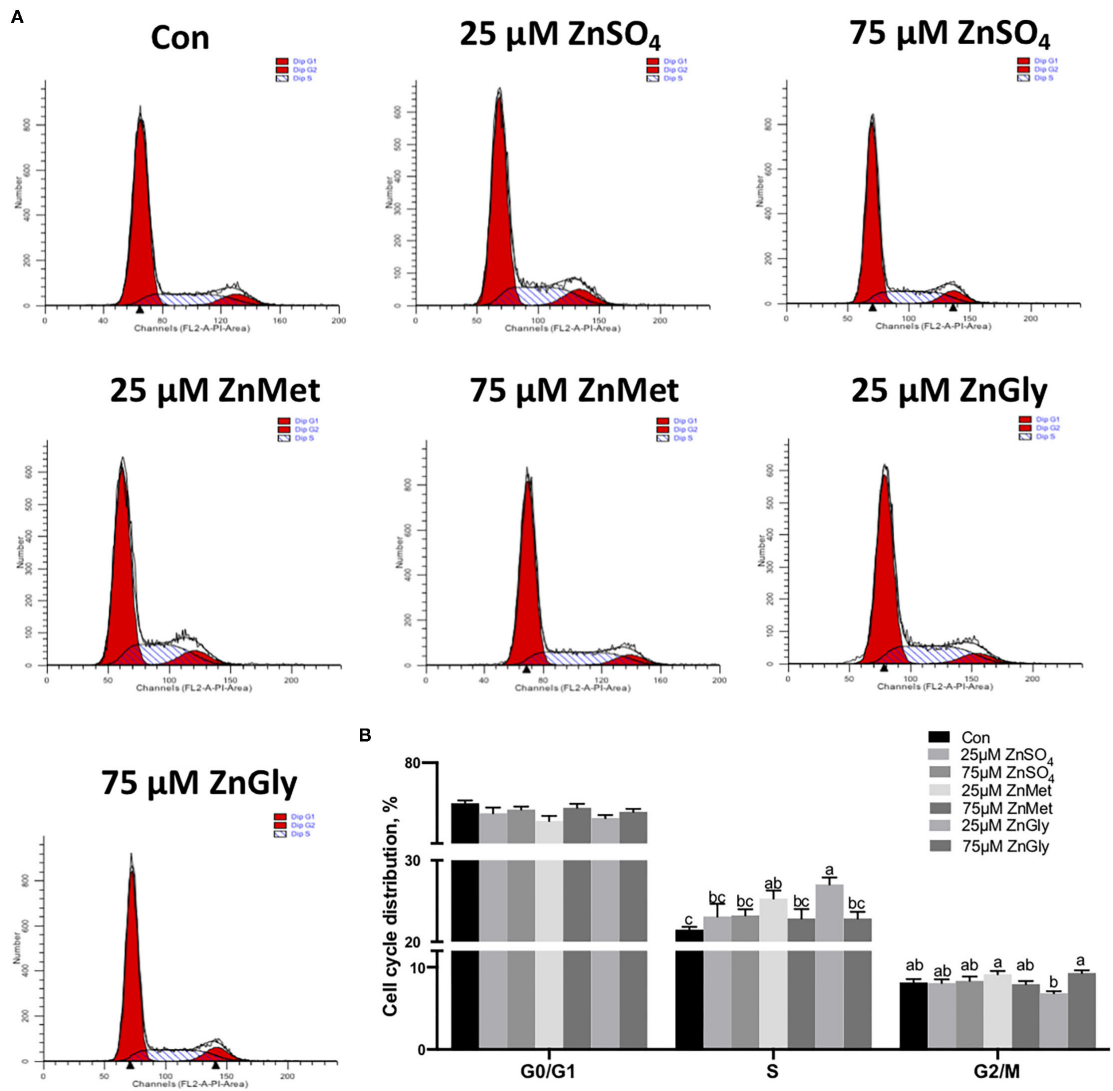
Cell cycle distribution **(A)** and its analyzation **(B)** of C2C12 cells by adding different zinc sources.

As the KEGG pathway was enriched in the cell cycle (ko04110) pathway, we determined the effect of ZnMet and ZnGly on the cell cycle. We treated the myoblasts with or without ZnSO_4_, ZnMet, and ZnGly (25 μM) for 48 h after serum starvation. The results showed that after 25 μM ZnMet and ZnGly treatment, there were more cells distributed in the S phase compared to the control (*P* < 0.05). Meanwhile, 25 μM ZnMet and 75 μM ZnGly treatments showed more cells distributed in G2/M phase compared to the 25 μM ZnGly treatment (*P* < 0.05). Compared to the control, no significant difference was noted in G0/G1 phase between the treatments (Figure 4).

### The Expression of the mTOR Pathway

**Figure 5:**
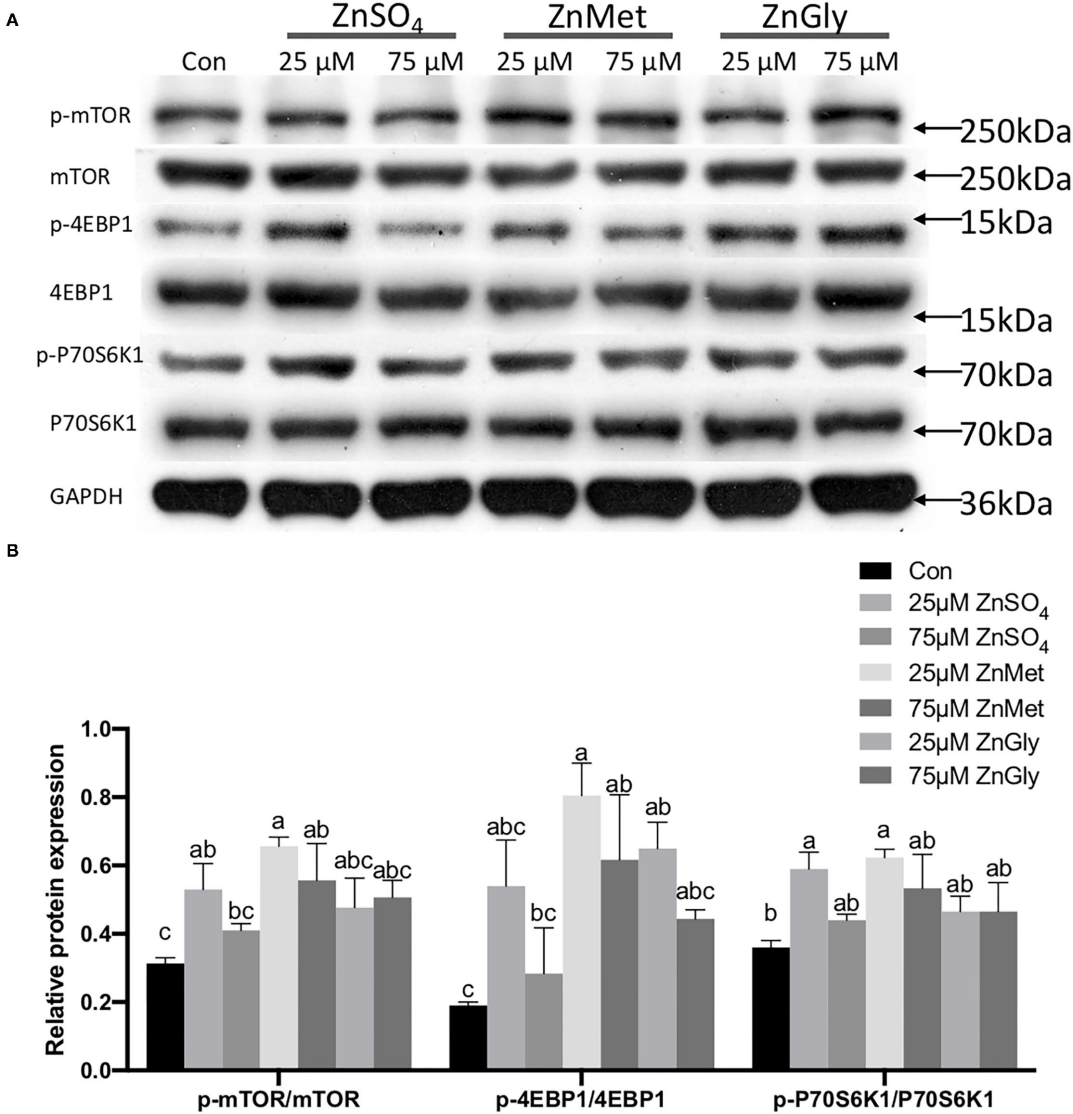
Phosphorylated protein expression levels of mTOR signaling pathway (mTOR, 4EBP1, and P70S6K1) **(A)** and the ratios of p-mTOR to mTOR, p-4EBP1 to 4EBP1, and p-P70S6K1–P70S6K1 **(B)** are regulated by adding different zinc sources.

As the KEGG pathway was enriched in the mTOR signaling pathway (ko04150), we measured the expression levels of the phosphorylated proteins of the key molecules in the mTOR signaling pathway and the results are shown in Figure 5A. Besides, the ratios of p-mTOR to mTOR, p-4EBP1 to 4EBP1, and p-P70S6K1–P70S6K1 were analyzed (Figure 5B). The results indicated that the protein expression levels of phosphorylated mTOR, 4EBP1, and P70S6K1 were all up-regulated in myotubes after treatment with 25 μM ZnMet (*P* < 0.05).

### Growth Characteristics and Carcass Traits of Growing-Finishing Pigs

The results of growth performance and carcass traits of growing-finishing pigs are detailed in Table 3. Compared to the ZnSO_4_ group, the ZnMet group showed an increase in the final BW (*P* < 0.05) and in ADG (*P* < 0.05). Besides, the ZnMet group showed a reduced tendency in the F/G ratio (*P* = 0.05). Carcass weight in the ZnMet group showed an increasing tendency when compared to the ZnGly group (*P* = 0.05). Meanwhile, the loin eye area in the ZnMet group showed an increasing tendency compared to the ZnSO_4_ groups (*P* = 0.05).

**Table 3:** Growth performance and carcass characteristics of finishing pigs fed Zn diets^a^.

**Item^**b**^**	**ZnSO_**4**_**	**ZnMet**	**ZnGly**	**SEM**	***P***
**Growth performance**					
Initial BW (kg)	31.63	31.65	31.58	0.36	1.00
Final BW (kg)	91.33^b^	97.92^a^	93.34^ab^	1.14	0.04
ADG (kg/d)	0.79^b^	0.88^a^	0.83^ab^	0.01	0.04
ADFI (kg/d)	1.94	1.98	2.11	0.04	0.12
F/G (feed/gain)	2.49^ab^	2.32^b^	2.55^a^	0.04	0.05
**Carcass characteristics**					
Carcass weight, kg	63.06^ab^	66.61^a^	62.23^b^	0.78	0.05
Dressing percentage, %	69.60	71.08	69.41	0.31	0.06
Loin eye area, mm^2^	35.07^b^	39.93^a^	37.41^ab^	0.82	0.05
Subcutaneous backfat depth, mm	19.67	21.01	18.08	1.03	0.54
PRA weight, kg	0.65	0.71	0.60	0.39	0.53

a *Different letters (a, b) within a row indicate the significant difference (P < 0.05), or a trend of difference (P = 0.05) among different treatments, n = 9*.

b *BW, body weight; ADG, average daily gain; ADFI, average daily feed intake; F/G, feed gain ratio; PRA, perirenal adipose tissue*.

Compared to the ZnSO_4_ group, the TP levels of ZnMet and ZnGly decreased (*P* < 0.05). Meanwhile, compared to the ZnSO_4_ group, the ALP level of ZnMet increased (*P* < 0.05; Table 4). There was no significant difference observed in the concentrations of ALB, BUN, NH_3_, GLU, TG, HDL-C, LDL-C, and CHOL among all of the treatments (*P* > 0.05).

**Table 4:** Serum metabolites of finishing pigs fed Zn diets^a^.

**Item^b^**	**ZnSO_**4**_**	**ZnMet**	**ZnGly**	**SEM**	***P***
TP, g/L	71.61^a^	69.55^b^	68.33^b^	0.46	0.01
ALB, g/L	46.23	44.31	43.11	0.59	0.09
ALP, U/L	114.43^b^	130.83^a^	127.29^ab^	2.30	0.04
BUN, mmol/L	4.34	3.78	4.06	0.10	0.08
NH_3_, μmol/L	143.02	141.93	144.79	3.07	0.94
GLU, mmol/L	5.58	5.53	5.48	0.08	0.89
TG, mmol/L	0.34	0.35	0.34	0.02	0.99
HDL-C, mmol/L	2.61	2.54	2.50	0.08	0.87
LDL-C, mmol/L	0.68	0.85	0.71	0.05	0.37
CHOL, mmol/L	1.77	1.65	1.71	0.08	0.83

a *Different letters (a, b) within a row indicate the significant difference (P < 0.05) among different treatments, n = 9*.

b *TP, total protein; ALB, albumin; ALP, alkaline phosphatase; BUN, blood urea nitrogen; NH_3_, ammonia; GLU, glucose; TG, triglyceride; HDL-C, high-density lipoprotein-cholesterol; LDL-C, low-density lipoprotein-cholesterol; CHOL, cholesterol*.

The results of mineral concentrations are summarized in Table 5. The ZnMet group showed a higher (*P* < 0.05) concentration of zinc in muscle, liver, kidney, spleen, and serum as compared to the ZnSO_4_ group. The ZnGly group also showed a higher (*P* < 0.05) concentration of zinc in the kidney as compared to the ZnSO_4_ group, but no significant difference was found in other tissues. Compared to the ZnSO_4_ group, the ZnMet group showed a higher (*P* < 0.05) concentration of iron in muscle, kidney, and serum, while the ZnGly group only showed a higher (*P* < 0.05) concentration of iron in the kidney. Meanwhile, The ZnMet group showed a lower (*P* < 0.05) concentration of copper in urine as compared to the ZnSO_4_ group. Collectively, compared to the ZnGly group, the ZnMet group showed relatively higher concentrations of zinc and iron in muscle, liver, kidney, spleen, and serum, and a lower concentration of copper in the urine.

**Table 5:** Micronutrients content of finishing pigs fed Zn diets^a^.

**Tissue**	**Item^b^**	**ZnSO_**4**_**	**ZnMet**	**ZnGly**	**SEM**	***P***
Muscle (mg/kg)	Zn	44.46^b^	51.08^a^	47.07^ab^	0.99	0.03
	Fe	21.52^b^	28.08^a^	21.56^b^	1.18	0.02
	Cu	2.50	2.94	3.39	0.15	0.10
Liver (mg/kg)	Zn	365.28^ab^	416.69^a^	348.43^b^	11.55	0.03
	Fe	725.53	795.02	773.94	35.57	0.73
	Cu	27.43	27.82	23.74	3.41	0.87
Kidney (mg/kg)	Zn	82.28^b^	108.84^a^	106.53^a^	4.41	0.02
	Fe	122.27^b^	154.69^a^	163.73^a^	6.40	0.01
	Cu	30.16	28.97	32.49	1.51	0.64
Heart (mg/kg)	Zn	78.38	79.91	77.01	1.61	0.77
	Fe	167.02	170.90	172.54	4.69	0.90
	Cu	15.85	18.15	15.47	0.79	0.33
Spleen (mg/kg)	Zn	83.05^b^	99.92^a^	89.76^b^	2.05	0.01
	Fe	829.45	1081.08	809.16	74.76	0.26
	Cu	1.75	2.16	2.16	0.10	0.14
Lung (mg/kg)	Zn	61.13	68.67	73.06	3.75	0.44
	Fe	358.30	384.30	322.49	25.23	0.62
	Cu	4.94	5.37	5.22	0.31	0.86
Serum (mg/L)	Zn	1.91^b^	3.13^a^	1.71^b^	0.22	0.01
	Fe	3.15^b^	5.10^a^	3.73^ab^	0.31	0.03
	Cu	2.74	2.96	2.63	0.07	0.15
Urine (mg/L)	Zn	2.31	1.48	1.98	0.20	0.26
	Fe	3.29	2.98	4.00	0.19	0.11
	Cu	0.91^a^	0.72^b^	1.01^a^	0.04	0.01

a *Different letters (a, b) within a row indicate the significant difference (P < 0.05) among different treatments, n = 9*.

b *Zn, zinc; Fe, iron; Cu, copper*.

## Discussion

Zinc is a crucial micronutrient for maintaining growth, reproduction, and metabolism (1, 26, 27). In the present study, the trials of myocytes and growing-finishing pigs both showed that, compared to inorganic zinc, amino acid-chelated zinc could not only increase biological efficacy but also improve muscle deposition and growth performance.

### Amino Acid-Chelated Zinc Supplementation Increased Cell Proliferation Mediated by the Cell Cycle Pathway

It is reported that zinc is an essential nutrient for proliferation, which may be caused by the regulation of the cell cycle (16, 28). The KEGG pathway enrichment analysis of DEGs revealed that the cell cycle pathways (ko04110) are most likely implicated in vigorous growth responses and metabolism in cells treated with amino acid-chelated zinc. The cell cycle consists of four phases: G1, S, G2, and M. The first three phases (G1, S, and G2) comprise interphase in which rapid cell growth, DNA replication, and the preceding step for cell division occur. The M phase refers to the mitotic phase, in which cell growth ceases and the parent cells are divided into daughter cells (29). In the current study, we found that the 25 mM ZnGly group and the 25 mM ZnMet group both drove more cells from G0/G1 phase to the S phase, while the 25 mM ZnMet group also drove more cells to the G2/M phase, indicating that most cells were proliferated into S and G2/M phase, thus increasing cell proliferation under the 25 mM ZnMet treatment. Besides, our CCK8 results also showed that amino acid-chelated zinc largely increased the viability of myoblasts, suggesting that amino acid-chelated zinc promotes C2C12 myoblast growth *via* the cell cycle pathway.

### Amino Acid-Chelated Zinc Enhanced Energy Metabolism and Promote Growth and Differentiation Mediated by ATP Produced by OXPHOS

**S**keletal muscle differentiation is an integrative and complex process involving the exit of myoblasts from the cell cycle to form multinucleated myotubes (20). MyoG (Myogenin) plays a key role in the regulation of the final stages of differentiation, and once the MyoG gene is lacking, the myoblasts are unable to fuse into multinucleated myofibers (30). Our results indicated that ZnMet significantly increased the expression levels of the marker gene MyoG, which is consistent with the results of RNA-seq, suggesting that amino acid-chelated zinc may also play key roles in skeletal muscle cell differentiation. In addition, cell cycle progressive pathways and differentiated pathways are tightly connected to pathways governing metabolic regulation, whose energy was mainly offered by OXPHOS, a complex process releasing the energy stored in nutrients (31). The KEGG pathways related to OXPHOS were significantly enriched in C2C12 cells treated with amino acid-chelated zinc in RNA-seq analysis.

When comparing different zinc sources, it was found that the bioavailability of organic zinc sources was relatively higher than that of inorganic zinc (8). In the present study, the final BW and ADG of pigs in the ZnMet group were higher than that in the ZnSO_4_ group, indicating a beneficial effect of amino acid-chelated zinc on growth performance and energy metabolism. Meanwhile, ZnMet group showed a relatively lower F/G than other groups, suggesting higher bioavailability of ZnMet. Moreover, in terms of carcass traits, ZnMet group also showed a relatively higher carcass weight, dressing percentage, and loin eye area, showing that ZnMet supplementation may lead to higher energy metabolism, protein synthesis, and muscle mass in pigs. Actually, in terms of energy metabolism, protein synthesis in the skeletal muscle is the major energy-consuming process, which becomes particularly essential when cells are committed to growth and proliferation (32). Therefore, energy supply plays a key role in the support of protein synthesis, which is mainly provided in the form of ATP (33). In the present study, cell growth in the C2C12 trial and skeletal muscle mass in the growing-finishing pigs are both linked to ATP availability, which was mainly produced by the OXPHOS pathway.

The impact of nutrition on metabolism is also directly reflected in the changes in the serum biochemical indicators. Serum TP is an important indicator of the level of protein metabolism in the liver (34). It is reported that serum TP level is inversely correlated with protein deposition in skeletal muscle tissues under the same intake of dietary nutrients (35). In the present study, the ZnMet group showed a significantly lower TP than the ZnSO_4_ group. Thus, lower TP in the ZnMet group rather suggests an increased deposition of protein in muscle tissue, which is in line with the effect on final **BW** and carcass weight, which were greater in the ZnMet group than in the ZnSO_4_ group. Besides, ALP is a zinc-containing metalloenzyme (36), thus it can be used as a marker of zinc status in the body (37). Meanwhile, it is reported that greater activity of serum ALP is conducive to energy metabolism and muscle mass (38). Intriguingly, the ZnMet group showed a significantly higher abundance of ALP, indicating a higher status of zinc concentration and muscle mass in the ZnMet group, which is as per the results of growth performance and carcass traits. In addition, serum ALB is a major zinc-transporting protein (39), and it is reported that organic zinc forms, such as ZnGly, could increase the level of ALB in growing pigs (37). However, the difference of ALB was non-significant among the treatments in this study, which may be detected in a further study. Moreover, the concentrations of TG, HDL-C, LDL-C, and CHOL in the serum reflect the dynamic lipid metabolic status of mammals (24). However, the serum levels of these lipid-related indicators in the ZnMet or ZnGly groups are not significantly different from those in the ZnSO_4_ group, indicating that the lipid metabolism is not greatly affected by the different zinc sources diets.

### Amino Acid-Chelated Zinc Increased Protein Metabolism and Muscle Deposition by the ATP Sensor, mTOR Pathway Zinc Sources on Growth Responses

When intracellular ATP concentrations change, mTOR functions as the homeostatic ATP sensor that plays a role in regulatory pathways that control ribosome biogenesis and cell growth (33). In response to mitogens and amino acids, mTOR phosphorylates and controls the activities of two key translational regulators, P70S6K1 and 4EBP1, subsequently enhancing translation initiation and protein synthesis (40). In the present study, the expression of mTOR and its two key downstream effectors (P70S6K1 and 4EBP1) in C2C12 were affected by different concentrations of different zinc sources, where the amino acid-chelated zinc performed better than the inorganic zinc. Among them, the group of 25 μM ZnMet performed the best, which is consistent with the KEGG analysis in RNA-seq.

### Amino Acid-Chelated Zinc Enhanced the Absorption and Utilization of Trace Elements

It is reported that ZnMet had a higher bioavailability among zinc sources (41); therefore, we further detected the concentration of zinc in serum and tissues, which can reflect the absorption and utilization of zinc and is also used as an indicator to evaluate the nutritional status of the body (42). An increase in serum zinc concentration indicates that zinc metabolism is enhanced (43). In this study, whether it is serum zinc or serum iron, the ZnMet group has the highest content, indicating that the addition of ZnMet improved the absorption of trace elements. Besides, the content of trace elements in the liver is relatively high and stable, which can reflect the level and deposition status of trace elements in the body to a certain extent. After a series of digestion processes, 67–80% of the zinc absorbed into the blood enters the liver and is first deposited in the liver, spleen, kidney, and only a small amount is deposited in muscles and other tissues (44). Therefore, each tissue has a different ability to accumulate zinc, resulting in different concentrations. In our study, the ZnMet group had the highest zinc and iron content in muscles and kidneys, indicating that ZnMet can improve the deposition of zinc and iron in these organs. In the liver and spleen, the zinc content of the ZnMet group was the highest, indicating that the addition of ZnMet can improve their absorption and utilization of micronutrients. Therefore, the addition of ZnMet showed a higher bioavailability, which is consistent with the results of growth performance.

## Conclusions

In conclusion, these findings provide new insights into the molecular and physiological mechanisms by which the amino acid-chelated zinc, especially ZnMet, improves myoblast growth while increasing bioavailability, which appears to be associated with the cell cycle, OXPHOS, and mTOR pathways, ultimately enhancing muscle mass. Specifically, compared to the inorganic zinc form, the dietary supplementation of 75 mg/kg amino acid-chelated zinc (ZnMet is better than ZnGly) could improve the growth performance and carcass traits of growing-finishing pigs. Furthermore, these findings also provide a theoretical basis and guidance for the future use of amino acid-chelated zinc to effectively replenish energy in animal nutrition and production.

## Data Availability Statement

The datasets presented in this study can be found in online repositories. The names of the repository/repositories and accession number(s) can be found at: https://www.ncbi.nlm.nih.gov/, PRJNA808668.

## Ethics Statement

The animal study was reviewed and approved by Animal Protection Committee of the Institute of Subtropical Agriculture, Chinese Academy of Sciences.

## Author Contributions

LZ, QG, and FL contributed to the study concept and critically reviewed the article. LZ, QG, YD, HN, CZ, and FL contributed to the design of the manuscript and figure preparation and edition. LZ, XL, QG, and FL contributed to the acquisition and analysis of data and drafted the manuscript. All authors gave final approval for all aspects of the work, agreed to be fully accountable for ensuring the integrity and accuracy of the work, read, and approved the final manuscript.

## Funding

This work was funded by the National Natural Science Foundation of China (31972582 and 32102572), the Science and Technology Program of Hunan Province (2021RC4039), Distinguished Young Scholar Fundation of Hunan Province (2020JJ2030), Key R&D Program of Hunan Province (2022NK2026), the Science and Technology Projects of Changsha City (kq1801059), the Youth Innovation Promotion Association CAS (Y202079), and the Earmarked Fund for China Agriculture Research System (CARS-35). Grant from the Innovation Team in Key Area Innovation Team of Physiology and Metabolism and Body Health in Pig (2019RS3022), Hunan Province Key Laboratory of Animal Nutritional Physiology and Metabolic Process (2018TP1031).

## Conflict of Interest

XL was employed by Guangzhou Tanke Bio-tech Co., Ltd. The remaining authors declare that the research was conducted in the absence of any commercial or financial relationships that could be construed as a potential conflict of interest.

## Publisher's Note

All claims expressed in this article are solely those of the authors and do not necessarily represent those of their affiliated organizations, or those of the publisher, the editors and the reviewers. Any product that may be evaluated in this article, or claim that may be made by its manufacturer, is not guaranteed or endorsed by the publisher.
