# Supplementary material for: Comparison of the Effects of Inorganic or Amino Acid-Chelated Zinc on Mouse Myoblast Growth in vitro and Growth Performance and Carcass Traits in Growing-Finishing Pigs
Source: Front Nutr. 2022 Apr 7;9:857393. doi: 10.3389/fnut.2022.857393 (PMC9021508; doi:10.3389/fnut.2022.857393)

**Table S1** Summary of raw data quality from RNA-Seq.

| Sample Name^1^ | Total Raw Reads (M) | Total Clean Reads (M) | Total Clean Bases (M) | Clean Reads Q20 (%) | Clean Reads Q30 (%) | Clean Reads Ratio |
| --- | --- | --- | --- | --- | --- | --- |
| control_1 | 69.32 | 69.32 | 6.93 | 97.89 | 90.18 | 100.00 |
| control_2 | 67.27 | 67.27 | 6.73 | 97.92 | 90.26 | 100.00 |
| control_3 | 69.28 | 69.28 | 6.93 | 98.00 | 90.66 | 100.00 |
| treat1_1 | 69.60 | 69.60 | 6.96 | 98.16 | 91.16 | 100.00 |
| treat1_2 | 64.62 | 64.62 | 6.46 | 98.03 | 90.69 | 100.00 |
| treat1_3 | 66.48 | 66.48 | 6.65 | 98.06 | 90.86 | 100.00 |
| treat2_1 | 69.27 | 69.27 | 6.93 | 97.86 | 90.08 | 100.00 |
| treat2_2 | 71.49 | 71.49 | 7.15 | 97.91 | 90.30 | 100.00 |
| treat2_3 | 71.40 | 71.40 | 7.14 | 97.85 | 90.03 | 100.00 |
| treat3_1 | 69.80 | 69.80 | 6.98 | 97.95 | 90.41 | 100.00 |
| treat3_2 | 68.59 | 68.59 | 6.86 | 98.01 | 90.56 | 100.00 |
| treat3_3 | 66.17 | 66.17 | 6.62 | 98.13 | 91.00 | 100.00 |

^1^Four treatments: control, the blank control; treat1, zinc sulfate (ZnSO_4_); treat2, methionine-chelated zinc (ZnMet); treat3, glycine-chelated zinc (ZnGly).

**Table S2** Clean reads aligned with the reference sequence.

| Sample Name^1^ | Total Clean Read (M) | Total Mapping Gene Ratio (%) | Uniquely Mapping Gene Ratio (%) |
| --- | --- | --- | --- |
| control_1 | 69.32 | 81.06 | 77.56 |
| control_2 | 67.27 | 82.51 | 78.7 |
| control_3 | 69.28 | 84.11 | 80.25 |
| treat1_1 | 69.6 | 83.79 | 79.69 |
| treat1_2 | 64.62 | 82.19 | 78.29 |
| treat1_3 | 66.48 | 80.43 | 76.74 |
| treat2_1 | 69.27 | 81.13 | 77.52 |
| treat2_2 | 71.49 | 81.13 | 77.49 |
| treat2_3 | 71.4 | 80.48 | 77.05 |
| treat3_1 | 69.8 | 83.28 | 79.47 |
| treat3_2 | 68.59 | 84.04 | 80.07 |
| treat3_3 | 66.17 | 83.85 | 79.94 |

^1^Four treatments: control, the blank control; treat1, zinc sulfate (ZnSO_4_); treat2, methionine-chelated zinc (ZnMet); treat3, glycine-chelated zinc (ZnGly).

**Fig. S1.** Pearson correlation of log_10_(FPKM+1) between 12 samples of differentiated C2C12 cells*,* including 4 treatments × 3 replicates. Four treatments: control, the blank control; treat1, zinc sulfate (ZnSO_4_); treat2, methionine-chelated zinc (ZnMet); treat3, glycine-chelated zinc (ZnGly). Blue indicates high correlations, and white indicates low correlations.


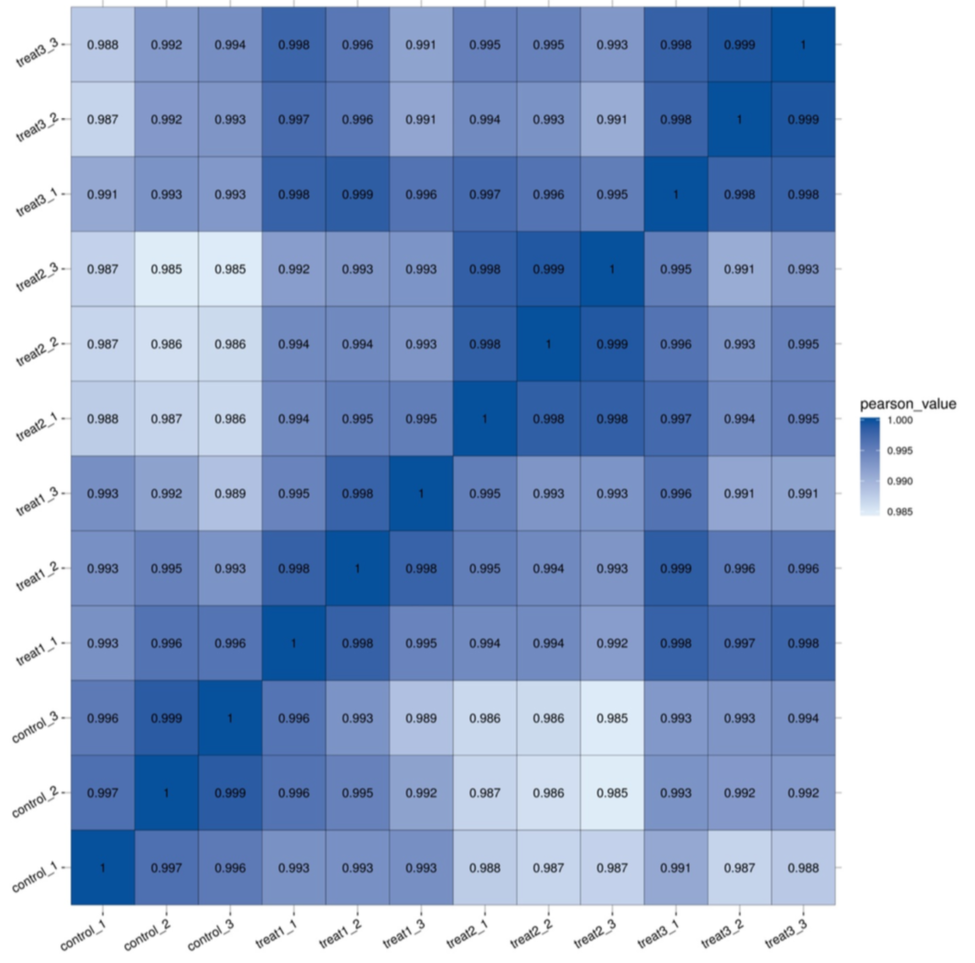

Supplement: Supplementary file 1 [file Table_1.DOCX]
